# Supplementary material for: Quantum Optical Immunoassay: Upconversion Nanoparticle-based Neutralizing Assay for COVID-19
Source: ArXiv. 2021 Oct 13:arXiv:2110.06755v1. Preprint. [Version 1] (PMC8528078)
Supplement: 1 [file NIHPP2110.06755V1-supplement-1.pdf]

Supplementary Materials: Quantum Optical Immunoassay: Upconversion Nanoparticle-based Neutralizing Assay for COVID-19

Navid Rajil[1,+,×,†,‡], Shahriar Esmaeili[1,+,†,‡], Benjamin W. Neuman[1,2,3,+,‡], Reed Nessler[1,+,‡], Hung-Jen Wu[4,+,‡], Zhenhuan Yi[1,+,‡], Robert W. Brick[1,+,‡], Alexei V. Sokolov[1,5,+,‡], Philip R. Hemmer[1,6,7,+,‡], and Marlan O. Scully[1,5,*,+,‡]

[1]Institute for Quantum Science and Engineering, Texas A&M university, TX 77843, US
[2]Department of Biology, Texas A&M University, College Station, TX 77843, US
[3]Global Health Research Complex, Texas A&M University, College Station, TX 77843, US
[4]Department of Chemical Engineering, Texas A&M University, College Station, TX 77843, US
[5]Baylor University, Waco, TX 76798, US
[6]Department of Electrical & Computer Engineering, Texas A&M University, College Station, TX 77843, US
[7]Zavoisky Physical-Technical Institute, Federal Research Center “Kazan Scientific Center of RAS”, Sibirsky Tract, 420029 Kazan, RU
[*]corresponding.scully@tamu.edu
[+]These authors conceptualized the experiment.
[×]This author built the microscope, prepared samples, acquired data.
[†]These authors processed the data.
[‡]These authors wrote the manuscript.

ABSTRACT

In a viral pandemic, a few important tests are required for successful containment of the virus and reduction in severity of the infection. Among those tests, a test for the neutralizing ability of an antibody is crucial for assessment of population immunity gained through vaccination, and to test therapeutic value of antibodies made to counter the infections. Here, we report a sensitive technique to detect the relative neutralizing strength of various antibodies against the SARS-CoV-2 virus. We used bright, photostable, background-free, fluorescent upconversion nanoparticles conjugated with SARS-CoV-2 receptor binding domain as a phantom virion. A glass bottom plate coated with angiotensin-converting enzyme 2 (ACE-2) protein imitates the target cells. When no neutralizing IgG antibody was present in the sample, the particles would bind to the ACE-2 with high affinity. In contrast, a neutralizing antibody can prevent particle attachment to the ACE-2-coated substrate. A prototype system consisting of a custom-made confocal microscope was used to quantify particle attachment to the substrate. The sensitivity of this assay can reach 4.0 ng/ml and the dynamic range is from 1.0 ng/ml to 3.2 μg/ml. This is to be compared to 19 ng/ml sensitivity of commercially available kits.

S1 Table of materials used

Table 1 shows the list of materials, company they were purchased from, and the part numbers.

Materials	Company and catalog number
Streptavidin coated UCNP	Creative diagnostics # DNLC041
Biotinylated receptor binding domain (RBD)	Acrobiosystems # SPD-C82E9
Bovine serum albumin (BSA)	Sigma-Aldrich # A7030
Tween 20	Sigma-Aldrich
10× PBS stock solution	Sigma-Aldrich # P5493-1L
Dopamine hydrochloride	Sigma-Aldrich # H8502
Tris HCl	Thermofisher #15568025
Nunc Labtek II 8-well bottom cover glass plates	Thermofisher # 155409
goat anti-mouse IgG with Alexa Fluor 633	Thermofisher # A-21052
Angiotensin-converting enzyme 2 (ACE-2)	Raybiotech # 230-30165
Recombinant SARS-CoV-2 RBD with C-terminal mouse IgG Fc Tag	Raybiotech # 230-30166
Mouse anti-SARS-CoV-2 neutralizing antibody clone NN54	Creative diagnostics # CABT-CS064
Human anti-SARS-CoV-2 neutralizing antibody clone T01KHu	Thermofisher # 703958
Human anti-SARS-CoV-2 non-neutralizing antibody but binding clone CR3022	Absolute antibodies # AB01680-10.0

Table S1 – Materials used in this work

S2 Experimental methods

S2.1 upconverting particles Phantom virion (UCPV) preparation

$NaYF_4$,Yb,Er@$NaYF_4$ upconversion nanoparticles (UCNPs) coated with streptavidin were purchased from Creative Diagnostics. The biotinylated RBD was purchased from Acrobiosystem. UCNPs coated with biotinylated RBD via streptavidin and biotin binding serves as phantom viruses. To prepare the phantom virus UCNPs, a 200 µl solution 0.5 mg/ml of UCNPs was mixed with 10 µl of 0.2 mg/ml biotinylated RBD and incubated for 1 hour on a shaker. Then the particles were washed 3 times (as described below) and resuspended in assay wash buffer (1×PBS, 0.5 % BSA, 0.1 % tween-20). After resuspension, a 0.4 µg/ml solution was prepared and sterile filtered and kept at 4 C until use.

S2.2 Particle wash protocol

To wash the phantom virus particles after conjugation of biotinylated RBD on to streptavidin coated UCNPs, the particles were centrifuged at 9000 g for 10 minutes. 180 µl of supernatant was removed and replaced with 180 µl of assay wash buffer (1×PBS, 0.5% BSA, 0.1% tween 20). Then the particles were resuspended and sonicated in bath sonicator at 60 W power for 10 minutes. The bath water was constantly changed every 2-3 minutes with fresh ice-cold water to keep the phantom virus particles cold. This process was repeated 3 times with one exception. For the last wash, after removing 180 µl of supernatant, only 170 µl of assay wash buffer was added to raise the phantom particles final volume to 200 µl (concentration 0.5 mg/ml). Then a volume of 5 ml of 0.4 µg/ml phantom particle solution was made and filtered using 0.2 µm cellulose acetate syringe filters. One must note that 0.5 to 1 ml of final solution will be lost in filtration step and this amount must be considered to prevent shortage of particle solution.

S2.3 ACE-2/polydopamine coating of the glass plates

ACE-2 proteins were coated onto glass substrates using the published polydopamine modification protocol1. Briefly, a 10 mM solution of Tris-HCl solution (PH = 8) was prepared and used to prepare a 2 mg/ml solution of dopamine hydrochloride. The solution then was mixed with 0.75 mg/ml ACE-2 protein solution at 1:1 volume ratio. The mixture was then plated on treated Nunc Labtek II 8-well bottom cover glass plates at 10 µl per well. The plates were incubated for 2 hours at room temperature in a humidity chamber to prevent drying. After 2 hours of incubation, each well was washed with 500 µl wash buffer (1×PBS, 0.5% BSA, 0.1%Tween 20) 4 times and incubated with 500 µl per well of blocking buffer (1×PBS, 5% BSA, 0.1%Tween 20) for 1 hour. After blocking, each well was washed with 500 µl of washing buffer 4 times. The plates were freshly prepared prior to use.

S2.4 Examination of ACE-2/polydopamine coating of the glass plates

To examine ACE-2/polydopamine coating, we used RBD with mouse IgG Fc tag (RBD–FC) to identify ACE-2. Briefly, the prepared plates were incubated with 250 µl of RBD–Fc at a concentration of 10 µg/ml in washing buffer for 1 hour. Then, we washed the plates 4 times with washing buffer before adding 250 µl of the secondary antibody goat anti-mouse IgG with Alexa Fluor 633 at concentration of 10 µg/ml to detect the RBD. For comparison, negative control plates without RBD–FC or secondary antibodies were prepared. Samples without blocking were evaluated as well. The assay structure of this experiment is shown in figure 1 (a-f). The interactions between UCPVs and the polydopamine/ACE-2 coated plates were evaluated as well. For these test, we prepared a solution of 10 µg/ml UCPV and coated a prepared plate with 290 µl of this solution. To test the nonspecific binding between polydopamine and UCPVs, we prepared another plate coated with

only polydopamine (mixed with 1× PBS instead of ACE-2 protein at 1:1 ratio). After 1 hour of incubation at room temperature, we washed the plates 4 times with wash buffer, and the plates were air dried at room temperature. Then, the fluorescent detection was conducted with the confocal microscope.

S2.5 Upconversion nanoparticle-based antibody neutralization assay (UNIK)

After preparation of UCPV, a dilution of 1 µg/ml was prepared. Seven vials of 300 µl of 0.4 µg/ml particles were separated and 10 µl of different dilutions of antibody solution in wash buffer were added to each vial such that each vial received only one dilution of antibody sample. The samples were incubated on a shaker for 1 hour. This step was done in parallel to blocking the step of the plate preparation. After incubation of UCPV and antibody and the blocking of the plates, the plates were washed 4 times and 300 µl of each UCPV sample was added to separate wells of the plates and incubated for 1 hour on tilt shaker. After this incubation, the wells were washed 5 times with wash buffer and imaged for particle count. The plates were stored at 4 C until imaging.

S2.6 Data acquisition and processing

S2.6.1 ACE-2 protein coating examination data acquisition and processing

As described in section S2.4, we prepared several samples to check the coating of ACE-2 protein on the glass coverslip plates. As described, the ACE-2-coated plates were coated with RBD tagged with mouse IgG Fc. The RBD was detected using goat anti mouse IgG conjugated with Alexa Fluor 633. To detect the fluorescence, a custom-made laser scanning confocal microscope was used. The schematic of the microscope is shown in figure S1 (supplementary materials). From each sample, spectra of 25 points from a 5 by 5 grid were collected and averaged. The excitation laser was a 638 nm laser, and the laser's output power was set to 10 mW for all measurements. The acquisition time was 1 second. The collected spectrum for each negative and positive control sample was averaged and plotted. The results are shown in Figure S4 b, d, f, and h.

S2.6.2 UNIK data acquisition and processing

Data acquisition for up-conversion based antibody neutralization kit (described in section S2.5) and UCPV specific and nonspecific binding to polydopamine/ACE-2 and polydopamine coatings respectively (section S2.4) were done with some differences relative to ACE-2 coating examination (section S2.4).

A multimode high-power laser was focused on each sample with a 50 µm diameter spot size using an oil immersion objective (Leica HCX Plan Apo 40×/1.25-0.75 OIL CS ¥/0.17/E objective). The input power to the objective was measured to be 300 mW (Figure S2, measured at point A). Each data point was scanned 10 times. Each scan was 145 µm by 145 µm and this area was imaged using a raster scan of 8 × 8 points. The fluorescent image from the particles was then reflected onto an ICCD camera (Starlight Xpress Trius Pro 674). The effective imaged area was 87 µm by 145 µm for the antibody titer tests. Each point of the 8 by 8 raster scan was integrated for 200 ms, resulting in acquisition time of 12.8 seconds per image. Each sample was imaged 10 times on a 2 by 5 grid. The step size for this grid was 500 µm. The scanning, imaging, and optical setup details can be found in Figure S2. Image data were saved and reconstituted in Mathematica using a custom-made code. The software was used to count UCPV foci in 10 fields of view per test per data point, and then summed and averaged over 3 repetitions to yield the particle counts. Thus, the error bars in Figure 3a and 3b in the main article show the fluctuation of number of particles counted for each data point across 3 repetitions

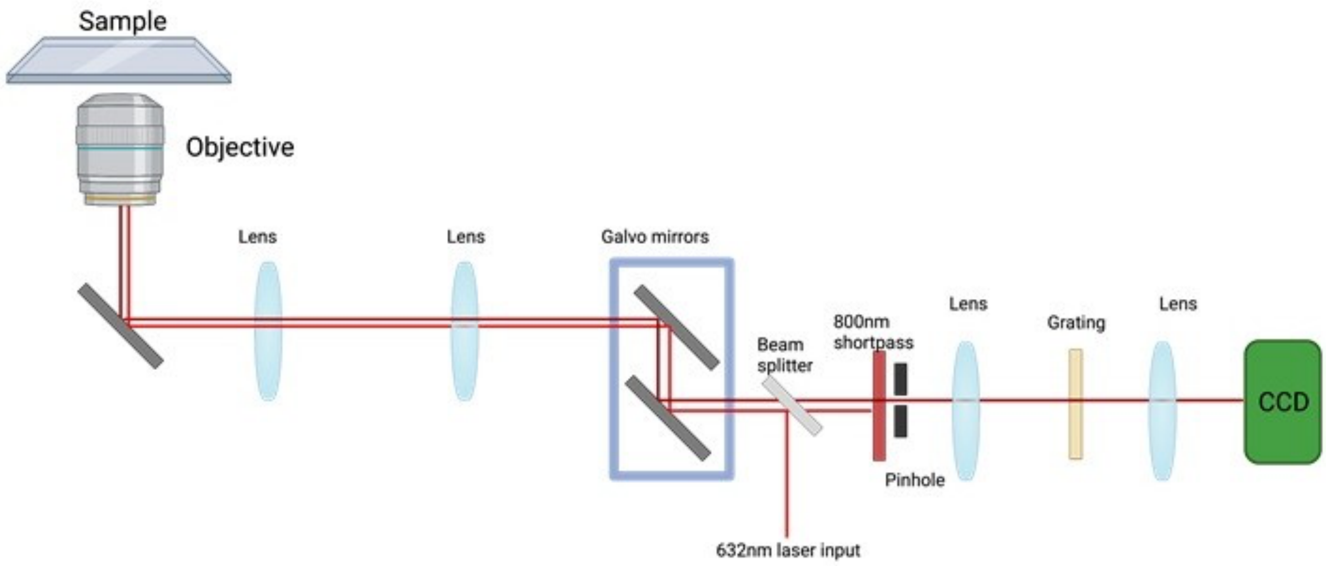

Figure S1 – Confocal spectroscopy and microscopy setup schematic. Using the galvo mirrors, the 632 nm laser is moved on the sample in a raster scan. A 632 nm notch filter (shown in red rectangle before pinhole) blocks the laser residual reflection. The image of the pinhole is blown through a transmission grating 300 lines/mm and imaged on an ICCD camera. The pixel counts are then transformed to spectrum data.

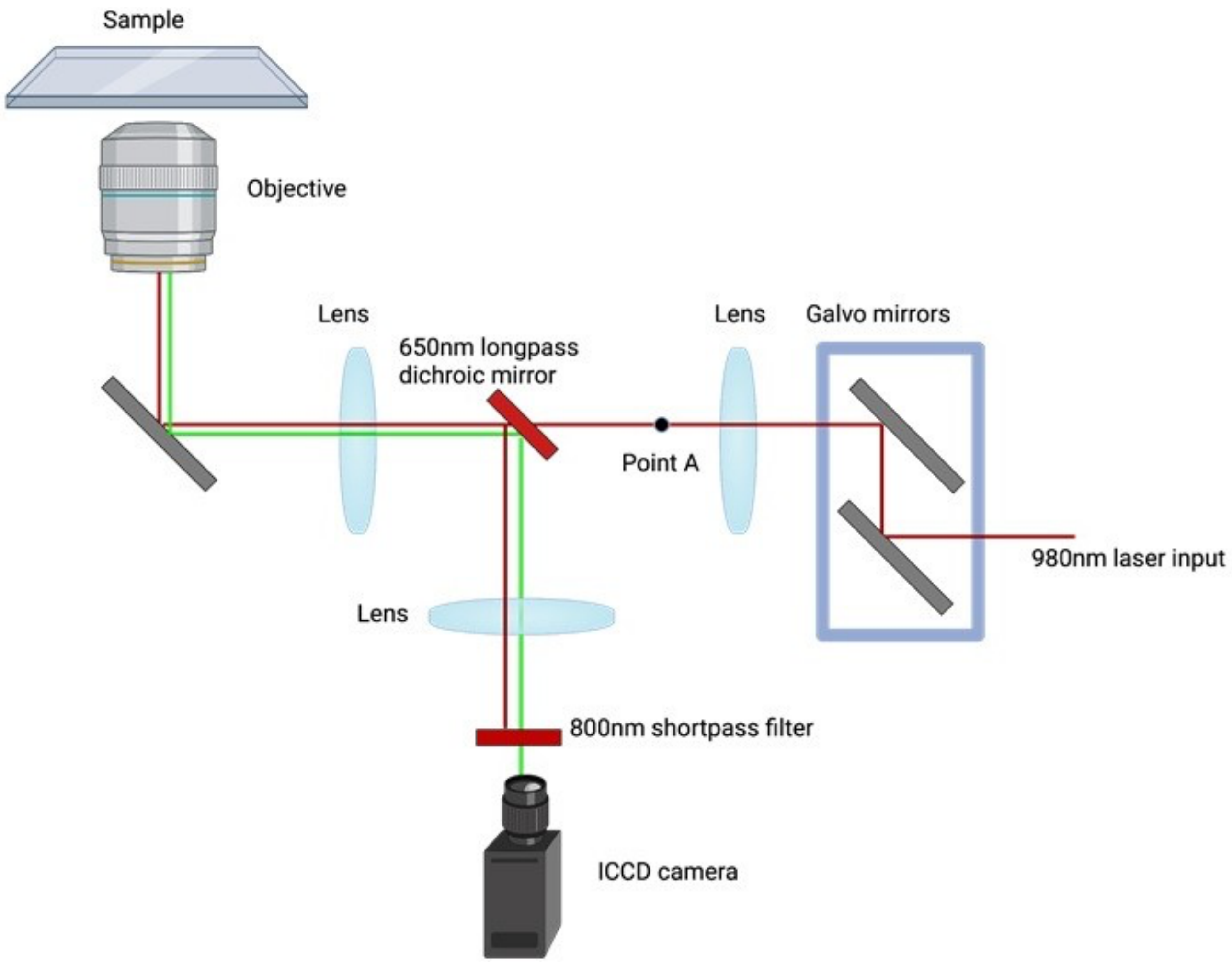

Figure S2 – Wide field fluorescence imaging setup schematic. Using galvo mirrors, the laser is moved on the sample in a raster scan. The ICCD camera collects the signal from each point of the raster scan during the whole time of the scan.

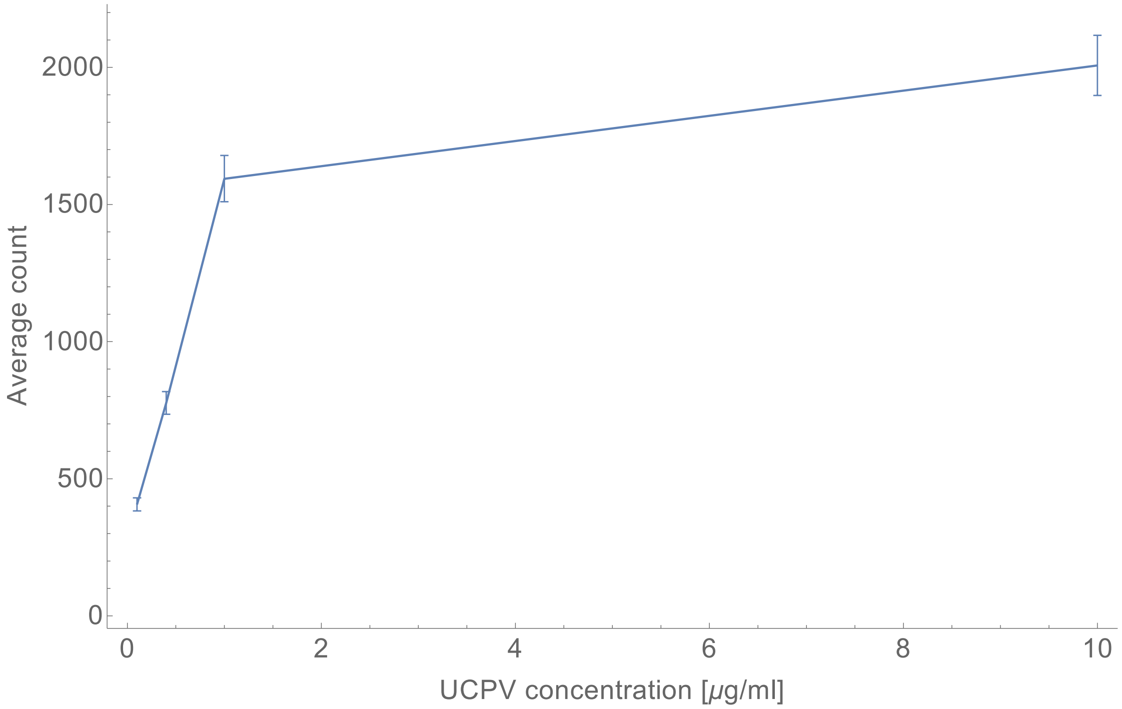

Figure S3 – Optimization of UCPV concentration. Average count per image of 5 images versus concentrations of UCPV at 0.1 μg/ml, 0.4 μg/ml, 1 μg/ml, and 10 μg/ml is plotted to choose optimized concentration of UCPV.

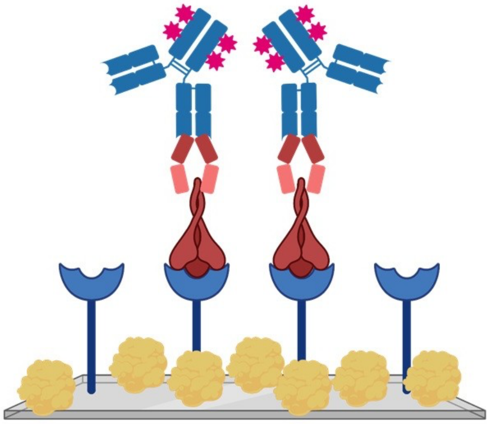

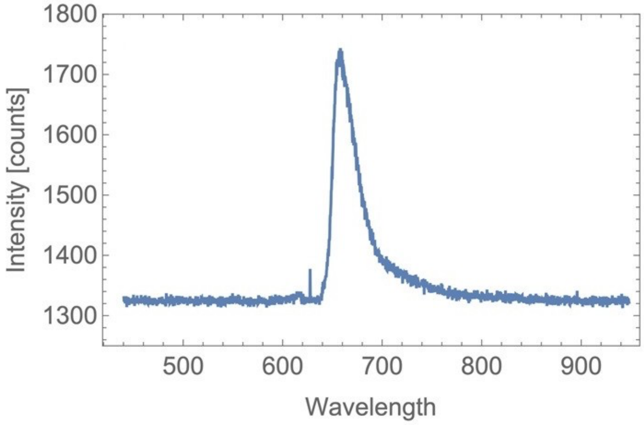

Intensity [counts]
1800
1700
1600
1500
1400
1300
500
600
700
800
900
Wavelength

(b)

(a)

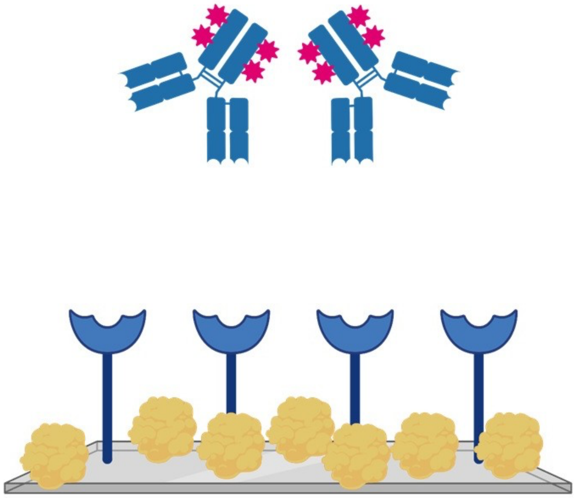

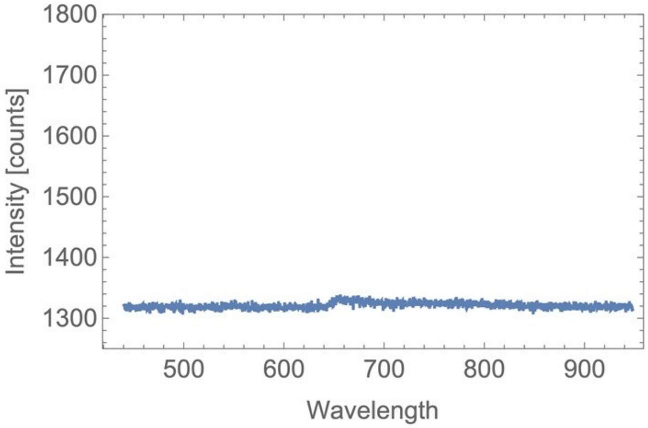

Intensity [counts]
1800
1700
1600
1500
1400
1300
500
600
700
800
900
Wavelength

(d)

(c)

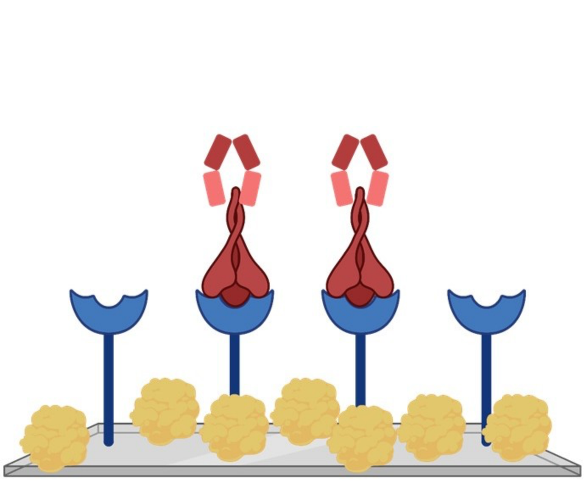

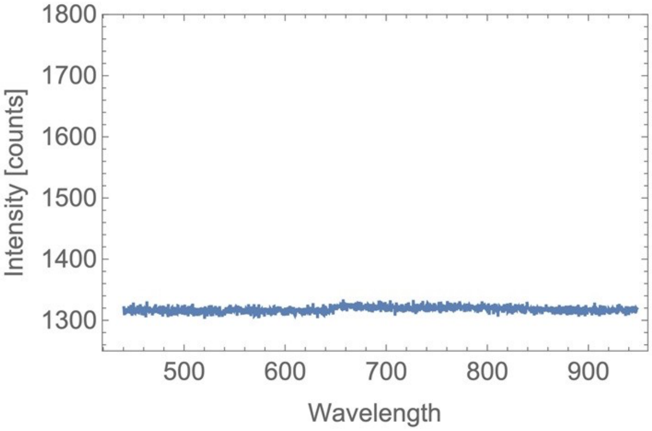

Intensity [counts]
1800
1700
1600
1500
1400
1300
500
600
700
800
900
Wavelength

(f)

(e)

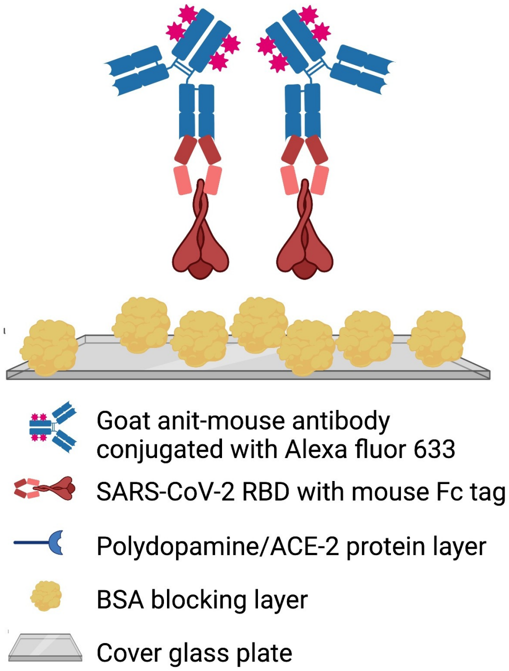

(g)

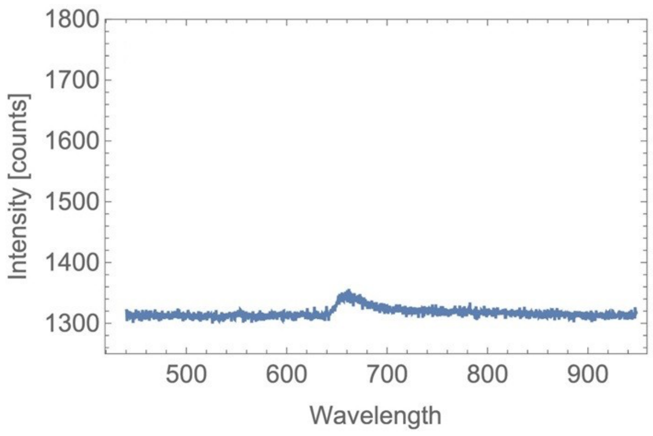

(h)

Figure S4 – ACE-2 coating test assay structure (a, c, e, g) and corresponding spectrum results (b, d, f, h). a) Full positive assay including ploydopamine/ACE-2 coating, blocking, RBD–FC, and the secondary antibody. c) Full negative control assay Missing RBD–FC. e) Positive assay missing secondary antibody to assess the autofluorescence background. g) Positive assay Missing ACE-2 protein (coated with polydopamine mixed with 1×PBS) to assess the non-specific background. b) Full positive assay including ploydopamine/ACE-2 coating, blocking, RBD–FC, and secondary antibody. A strong fluorescence from secondary antibody is observed. d) Full negative control assay Missing RBD–FC. A zero-fluorescence signal is expected and observed. f) Positive assay Missing secondary antibody to assess the autofluorescence background. No auto fluorescence background is observed. h) Positive assay Missing ACE-2 protein (coated with polydopamine mixed with 1× PBS) to assess the non-specific background in which a very weak signal was observed. Since 2c shows no fluorescence, considering assay structure, this signal most probably is due to nonspecific binding between RBD–FC and the plate.

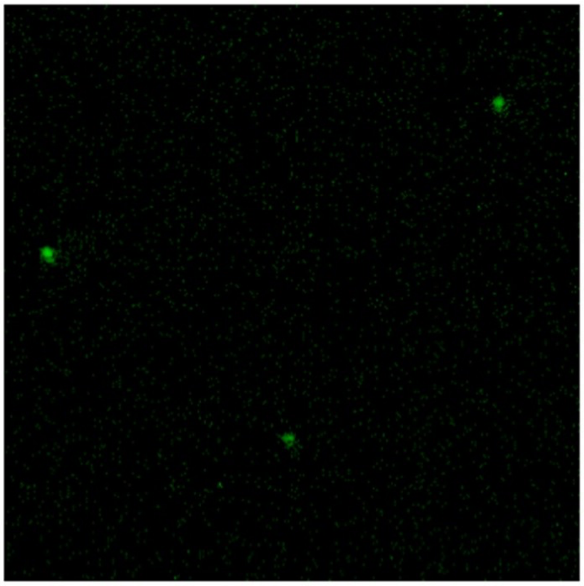

(a)

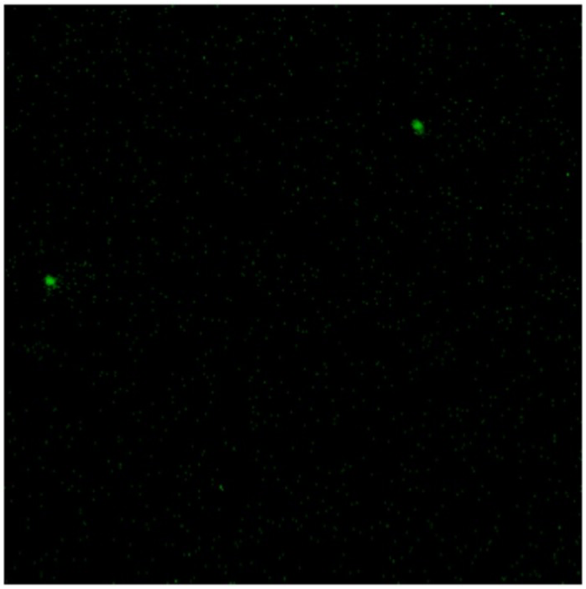

(b)

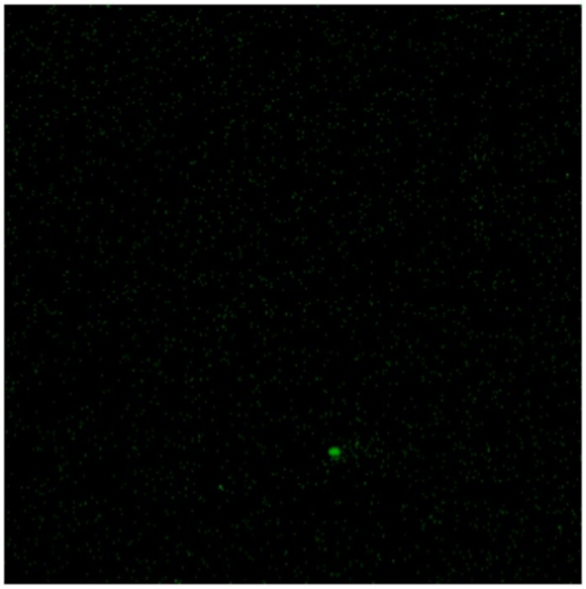

(c)

Figure S5 – Affinity of UCPV and Polydopamine/PBS coated area. a, b, c) shows 3 scans of 3 different areas in the center of circular area coated with polydopamine/PBS to assess the nonspecific binding between UCPVs and blocked polydopamine. Small green spots are visible in each figure and represent a particle. This shows that the binding shown in Figure 2b is due to the intrinsic affinity between ACE-2 protein and RBD. All images are 53 μm by 53 μm.

S3 4-parameter logistic function fits

The 4-parameter logistic curves were fitted using the online tool available at ATT Bioquest[2]. For neutralizing antibody type 1 (NN54), the equation, IC50 (midpoint), and Hill coefficient are below:

$$Y_{NN54} = B + \frac{A - B}{1 + (con_{NN54}/IC50)^{hc}} \quad (S1)$$

where $A = 7123$, $B = 3603$, $IC50 = 0.0122$, $hc = 1.148$, and con_{NN54} is the concentration of antibody (clone NN54). So:

$$IC50 = 0.0122\ \mu g/ml \text{ and Hill coeff.} = 1.148 \quad (S2)$$

For neutralizing antibody type 2 (T01KHu), the equation, IC50 (midpoint), and Hill coefficient are shown below:

$$Y_{T01KHu} = B + \frac{A-B}{1+(con_{T01KHu}/IC50)^{hc}} \quad (S3)$$

where $A = 6892$, $B = 4035$, $IC50 = 0.138$, and $hc = 4.084$, and con_{T01KHu} is the concentration of antibody (clone T01KHu). So,

$$IC50 = 0.138\ \mu g/ml \text{ and Hill coeff.} = 4.084 \quad (S4)$$

S4 Limit of detection (LOD) calculation

The standard deviation for NN54 negative control sample was 423 counts. Thus, using S1:

$$x_{LOD}^{NN54} = 0.004\ \mu g/ml \quad (S5)$$

The standard deviation for T01KHu negative control sample was 608 counts. Thus, using S3:

$$x_{LOD}^{T01KHu} = 0.128\ \mu g/ml \quad (S6)$$

S5 Calculated *p*-values for each data point

To calculate the P value, we performed the T-test using the built-in function in Mathematica software. Total counts of each data point (set of 3 numbers from the 3 repetitions) were compared with the set of total counts negative control (data point with no antibody) data set.

Antibody type	*p*-value 0.00323 μg/ml	*p*-value 0.0323 μg/ml	*p*-value 0.0968 μg/ml	*p*-value 0.194 μg/ml	*p*-value 0.323 μg/ml	*p*-value 3.23 μg/ml
NN54 (neutralizing type1)	NS	3.4×10^{-3}	6.5×10^{-4}	3.3×10^{-4}	3.5×10^{-4}	1.8×10^{-4}
T01KHu (neutralizing type2)	NS	NS	NS	6.5×10^{-3}	5.5×10^{-3}	3.0×10^{-3}
CR3022 (Non-neutralizing type3)	NS	NS	NS	NS	NT	NS

Table S2 – *p*-value of total counts of 3 repetitions of each data point for each antibody calculated against the set of 3 repetition of negative control counts. One star when *p*-value ≤ 0.05, two stars when *p*-value ≤ 0.01, three stars when *p*-value ≤ 0.005, and four stars when *p*-value ≤ 0.001. NT stands for not tested. NS stands for non-significant.

S6 KD value and θ approximation

This difference in IC50 also provides evidence for the inherent assay sensitivity which is limited by the affinity between antibody and antigen[3]. For instance, in the equation below for protein P binding with ligand L and producing protein–ligand complex PL:

$$P + L \rightleftharpoons PL \tag{S7}$$

The dynamic equation for the concentration of protein–ligand complex $[PL]$ can be written as

$$\frac{d[PL]}{dt} = \kappa_a[P]_f[L]_f - \kappa_d[PL] \tag{S8}$$

Where κ_a is the association rate of protein and ligand, κ_d is the dissociation rate of protein–ligand complex, $[P]_f$ and $[L]_f$ are free protein and free ligand concentration. At equilibrium this equation is equal to zero $\frac{d[PL]}{dt} = \kappa_a[P]_f[L]_f - \kappa_d[PL]_f = 0$, which leads to:

$$\frac{[P]_f[L]_f}{[PL]} = \frac{\kappa_d}{\kappa_a} \equiv K_d. \tag{S9}$$

In a simple case, we can define the ratio of proteins-ligand concentration to total protein as:

$$\theta = \frac{[PL]}{[P]_t} \tag{S10}$$

with

$$[P]_f = [P]_t - [PL] \tag{S11}$$

where $[P]_t$ is the total concentration of protein. Substituting S11 in S9 and approximate $[L]_f = [L]_t$ (leading to the higher bound for θ) and rearranging will yield

$$\frac{([P]_t - [PL])[L]_t}{[PL]} \approx K_d \tag{S12}$$

Simple algebra and rearranging will yield

$$\theta = \frac{[PL]}{[P]_t} \approx \frac{[L]_t}{[L]_t + K_d} \tag{S13}$$

The parameter θ is the ratio of filled proteins and is related to K_d. θ = 0.5 when $[L]_t = K_d$. So, in some sense K_d value (reported in Molar) is the concentration of ligand at which 50% of the proteins are filled with ligands. Now, since one basicall measures the number of filled proteins on the substrate in an ELISA-based assay (through any means of measurement), then it is easy to see that (as an approximation):

$$\theta = \frac{[PL]}{[P]_t} \approx \frac{[L]_t}{[L]_t + K_d} = 0.5 \text{ and is proportional to} IC50 \tag{S14}$$

Thus, one can correlate IC50 point to the K_d value.

As such, the best strategy to improve the limit of detection (LOD) for a certain antigen is to first acquire the best antibody with lowest dissociation constant (K_d value) and implement the detection apparatus capable of achieving said sensitivity. In general, since K_d value of antibodies for their targets varies between 10^{-5} M to 10^{-12} M from antibody to antibody, the detection assay's LOD will vary from antibody to antibody.

S7 Exact solution for θ

$$\frac{[P]_f[L]_f}{[PL]} = K_d \tag{S15}$$

$$[P]_f + [PL] = [P]_t \tag{S16}$$

$$[L]_f + [PL] = [L]_t \tag{S17}$$

Using S16 and S17 in S15 and simple algebra will yield

$$[P]_t[L]_t - ([P]_t + [L]_t + K_d)[PL] + [PL]^2 = 0 \tag{S18}$$

Solving S18 for [PL] gets

$$[PL]_1 = \frac{([P]_t + [L]_t + K_d) - \sqrt{([P]_t + [L]_t + K_d)^2 - 4[P]_t[L]_t}}{2} \tag{S19}$$

$$[PL]_2 = \frac{([P]_t + [L]_t + K_d) + \sqrt{([P]_t + [L]_t + K_d)^2 - 4[P]_t[L]_t}}{2} \tag{S20}$$

Assuming a constant concentration for $[P]_t$ and only titer $[L]_t$, the second solution is unphysical since $\lim_{[L]_t\ \infty}[PL]_2 = \infty$ is not bounded. Thus only $[PL]_1$ is an acceptable solution. Thus:

$$\theta = \frac{[PL]}{[P]_t} = \frac{([P]_t + [L]_t + K_d) - \sqrt{([P]_t + [L]_t + K_d)^2 - 4[P]_t[L]_t}}{2[P]_t} \tag{S21}$$

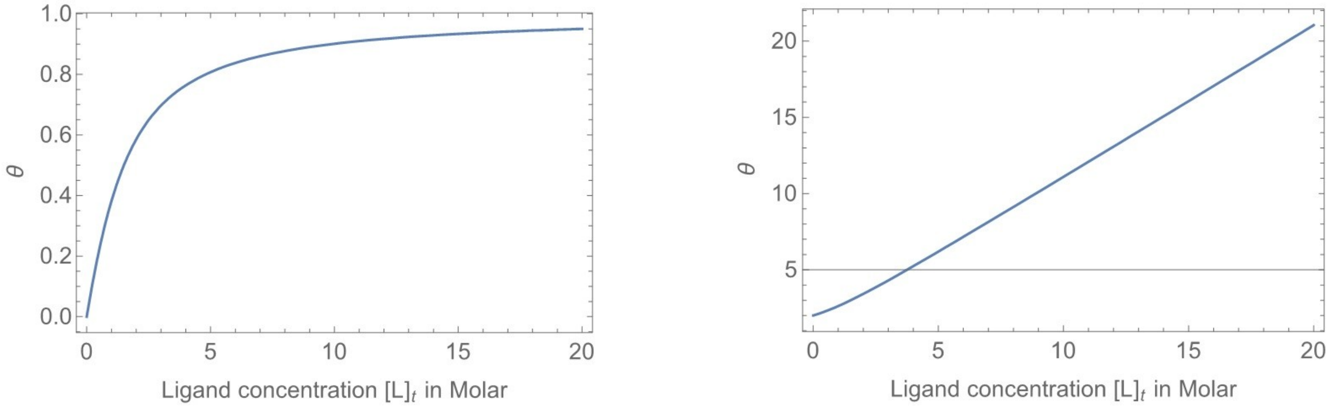

Figure S6 – a) Plot of $[PL]_1$ for the case of K_d = $[P]_t$ = 1. b) Plot of $[PL]_2$ for the case of K_d = $[P]_t$ = 1.

S8 The Theoretical solution for UNIK

The counted particles in the image are those that have bound to the ACE-2 protein on the substrate. Thus, we can write θ_1 for this binding as

$$\theta_1 = \frac{[ACE.UCPV]}{[ACE]_t} = \frac{([ACE]_t + [UCPV]_f + K_d^{(1)}) - \sqrt{([ACE]_t + [UCPV]_f + K_d^{(1)})^2 - 4[ACE]_t[UCPV]_f}}{2[ACE]_t} \tag{S22}$$

It should be noted that the $[UCPV]_f$ here is the total available UCPVs (or UCPVs) that are not blocked by the antibodies. Thus, we must find the concentration of non-blocked RBDs. We can find the ratio of blocked RBDs using S24.

$$\theta_2 = \frac{[UCPV.Ab]}{[UCPV]_{total}} = \frac{([UCPV]_{total} + [Ab]_t + K_d^{(2)}) - \sqrt{([UCPV]_{total} + [Ab]_t + K_d^{(2)})^2 - 4[UCPV]_{total}[Ab]_t}}{2[UCPV]_{total}} \quad (S23)$$

Now, the total concentration of unblocked RBDs is $1 - \theta_2$

$$1 - \theta_2 = \frac{[UCPV]_f}{[UCPV]_{total}} = (([UCPV]_{total} - [Ab]_t - K_d^{(2)}) + \sqrt{([UCPV]_{total} + [Ab]_t + K_d^{(2)})^2 - 4[UCPV]_{total}[Ab]_t})/(2[UCPV]_{total}) \quad (S24)$$

Then

$$\frac{[UCPV]_f}{[UCPV]_{total}} = (([UCPV]_{total} - [Ab]_t - K_d^{(2)}) + \sqrt{([UCPV]_{total} + [Ab]_t + K_d^{(2)})^2 - 4[UCPV]_{total}[Ab]_t})/(2[UCPV]_{total}) \quad (S25)$$

$$[UCPV]_f = (([UCPV]_{total} - [Ab]_t - K_d^{(2)}) + \sqrt{([UCPV]_{total} + [Ab]_t + K_d^{(2)})^2 - 4[UCPV]_{total}[Ab]_t})/(2) \quad (S26)$$

This is the available RBD concentration that can bind to the substrate and be counted. Figure S6 shows the plot of q_1 when S27 is plugged in, as function of total antibody concentration [Ab]t. The parameter for this plot were $[RBD]_{initial} = [ACE]_t = K_d^{(1)} = K_d^{(2)} = 1$. It is evident that the LOD depends on ACE-2 concentration, initial RBD (UCPV) concentration, and K_d values of binding between RBD and antibody ($K_d^{(2)}$) and RBD and ACE-2 ($K_d^{(2)}$) protein. Only two can be controlled by assay developer, ACE-2 concentration and RBD (UCPV) concentration.

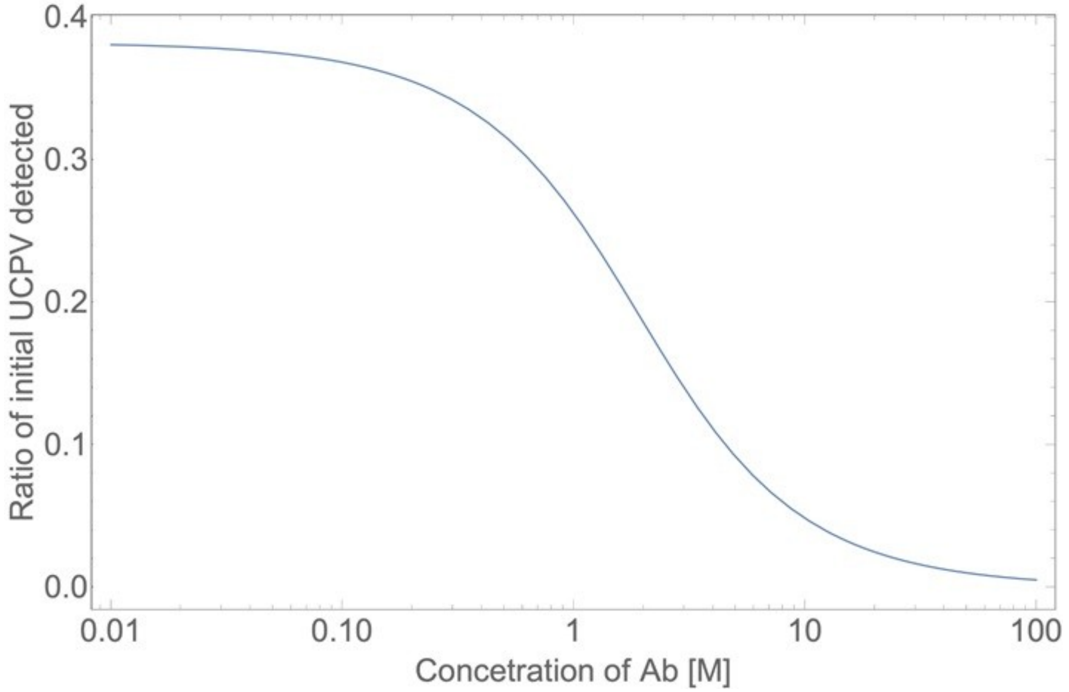

Figure S7 – Theoretical calibration plot of UNIK when $[UCPV]_{total} = [ACE]_t$ $[ACE]_t = K_d^{(1)} = K_d^{(2)} = 1$.

S9 Derivation $[Ab]_t|_{IC50}$ for UNIK

From S22 we can find the $[UCPV]_f|_{IC50}$ when 50% of ACE protein on the substrate is full, with simple algebra as

$$[UCPV]_f|_{IC50} = \frac{1}{2}[ACE] + K_d^{(1)}. \tag{S27}$$

Plugging in the S26 we get (note that $[UCPV]_f|_{IC50}$ happens at a certain antibody concentration which we show it as $[Ab]_t|_{IC50}$)

$$\frac{([UCPV]_{total} - [Ab]_t|_{IC50} - K_d^{(2)}) + \sqrt{([UCPV]_{total} + [Ab]_t|_{IC50} + K_d^{(2)})^2 - 4[UCPV]_{total}[Ab]_t|_{IC50}}}{2} = \frac{1}{2}[ACE] + K_d^2. \tag{S28}$$

Solving for $[Ab]_t|_{IC50}$ will yield

$$[Ab]_t|_{IC50} = [UCPV]_{total} - K_d^{(2)} + \frac{2[UCPV]_{total}K_d^{(2)}}{[ACE] + 2K_d^{(1)}} - \frac{1}{2}[ACE] - K_d^{(1)}. \tag{S29}$$

There are two basic assumptions inherit inS33. One is $[\mathrm{UCPV}]_{total} \neq 0$ and$[\mathrm{ACE}]_{total} \neq 0$. The second is the fact that$[Ab]_t|_{IC50} > 0$. This second condition will give us the following constraint:

$$[UCPV]_{total} > \frac{1}{2}[ACE] + K_d^{(1)}. \tag{S30}$$

With $K_d^{(1)} = 10^{-9}$ M, and $K_d^{(2)} = 10^{-12}$ M we will get the following graph for the general behavior of $[Ab]_t|_{IC50}$ as a function of [ACE] for different $[\mathrm{UCPV}]_{total}$ concentrations which shows that we must decrease UCPV concentration and maximize ACE-2 protein concentration

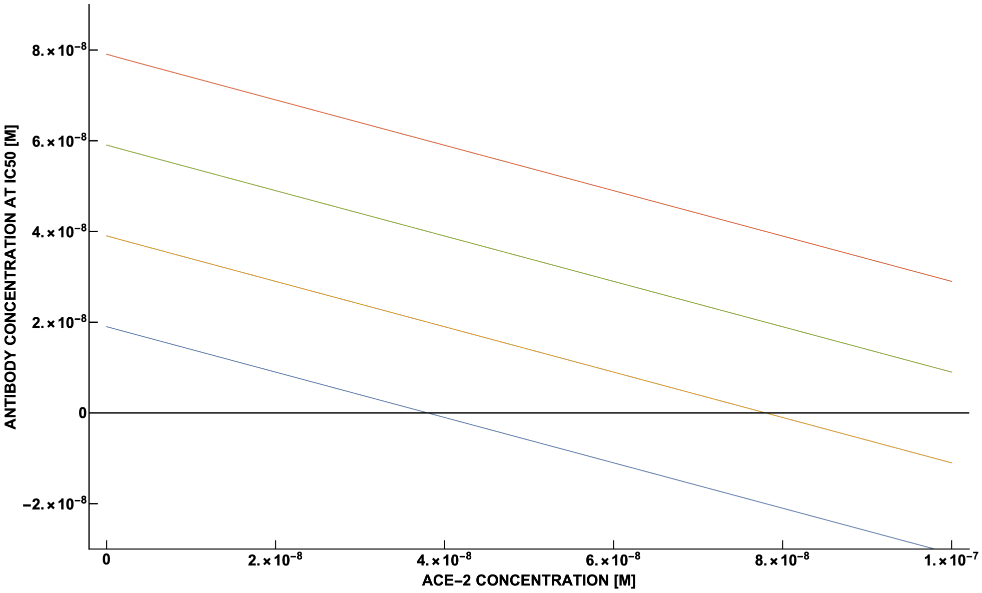

Figure S8 – Behavior of $[Ab]_t|_{IC50}$ as a function of ACE-2 concentration for different concentrations of $[UCPV]_{total}$. Red, green,, orange, and blue indicate $[UCPV]_{total} = 8 \times 10^{-8}$, $[UCPV]_{total} = 6 \times 10^{-8}$, $[UCPV]_{total} = 4 \times 10^{-8}$, $[UCPV]_{total} = 2$ 10^{-8} molar concentrations respectively. Other constants were $K_d^{(1)} = 10^{-9}$ M and $K_d^{(2)} = 10^{-12}$ M.

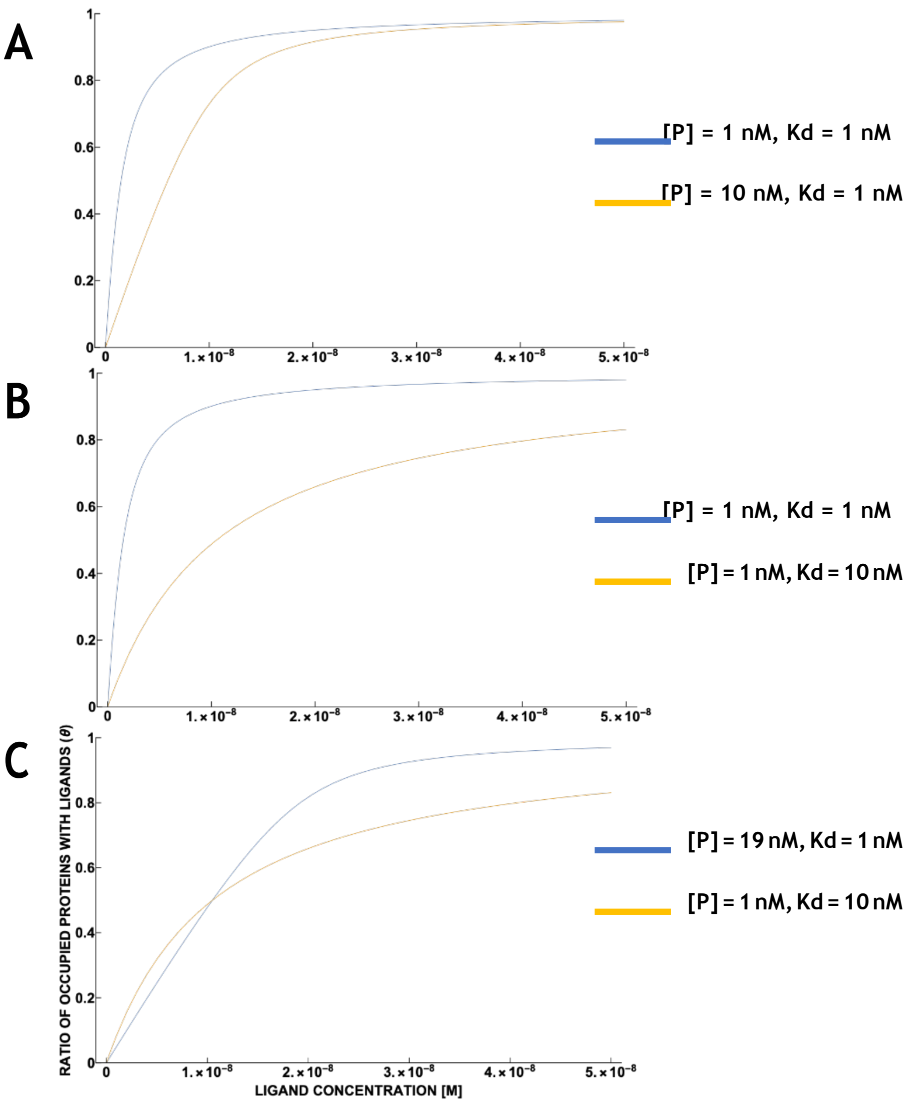

Figure S9 – Numerical solutions of θ in Eq. 2 for A) $[P]_t = 1nM$, $K_d = 1nM$ vs $[P]_t = 10nM$, $K_d = 1nM$. B) $[P]_t = 1nM$, $K_d = 1$ vs $[P]_t = 1nM$, $K_d = 10nM$, and C) $[P]_t = 19nM$, $K_d = 1nM$ vs $[P]_t = 1nM$, $K_d = 10nM$.

S10 A quantum description of θ

We consider a bimolecular reaction $A + B \rightleftharpoons AB$ where the initial populations of A and B are a and b, respectively. The population n of AB is modeled probabilistically as a birth–death process[4], where the birth rate from state n to $n+1$ is $\lambda_n = \alpha(a-n)(b-n)$ and the death rate from n to $n-1$ is $\mu_n = \beta n$. Here α and β are rate constants particular to the reaction. Using p_n ($n = 0,1,...,\min(a,b)$) to denote the probability distribution of n, we have the governing equations

$$\frac{dp_0}{dt} = -\lambda_0 p_0 + \mu_1 p_1 \qquad \text{(S31)}$$

$$\frac{dp_{0n}}{dt} = -\lambda_n p_n + \lambda_{n-1} p_{n-1} - \mu_n p_n + \mu_{n-1} p_{n-1} \text{ when } n \geq 1 \qquad \text{(S32)}$$

whose steady-state ($\frac{dp_n}{dt} = 0$) solution is

$$p_n = p_0 \prod_{k=1}^{n} \lambda_{k-1}/\mu_k \qquad \text{(S33)}$$

with p_0 determined by the normalization $\sum_n p_n = 1$. With the given birth and death rates this distribution is described by the probability-generating function

$$G(z) = \sum_{n=0}^{\min(a,b)} p_n z^n = \frac{{}_2F_0(-a,-b;;\frac{\alpha}{\beta}z)}{{}_2F_0(-a,-b;;\frac{\alpha}{\beta})} \tag{S34}$$

where ${}_2F_0$ is a generalized hypergeometric function. The expected population of AB at equilibrium is

$$G'(1) = ab\frac{\alpha}{\beta}\frac{({}_2F_0(1-a,1-b;;\frac{\alpha}{\beta})}{{}_2F_0(-a,-b;;\frac{\alpha}{\beta})}. \tag{S35}$$

When evaluated numerically this expression is found to agree closely with the non-probabilistic treatment of the law of mass action, i.e. where the equilibrium value of n solves the equation

$$((a-n)(b-n))/n = \beta/\alpha, \tag{S36}$$

which resembles Eqn. S15.

References

1. Moon, J. *et al.* Surface-independent and oriented immobilization of antibody via one-step polydopamine/protein g coating: Application to influenza virus immunoassay. *Macromol. Biosci.* **19**, 1800486, DOI:https://doi.org/10.1002/mabi.201800486 (2019).
2. AAT Bioquest, I. Quest graph™ four parameter logistic (4pl) curve calculator (2021).
3. Hulme, E. C. & Trevethick, M. A. Ligand binding assays at equilibrium: validation and interpretation. *Br. journal pharmacology* **161**, 1219–1237, DOI:10.1111/j.1476-5381.2009.00604.x(2010).
4. Feller, W. An introduction to probability theory and its applications. *1957* .
